# The Variant Allele Frequency of CTLA-4 rs11571317 (−658 C/T) Polymorphism With Colorectal Cancer Susceptibility in the Saudi Population and Other Ethnic Groups

**DOI:** 10.7759/cureus.50091

**Published:** 2023-12-07

**Authors:** Mohammad Salman Akhtar

**Affiliations:** 1 Department of Basic Medical Sciences, Faculty of Applied Medical Sciences, Al Baha University, Al Baha, SAU

**Keywords:** cancer, single nucleotide polymorphism, −658 c/t, rs11571317, ctla-4

## Abstract

The single nucleotide polymorphisms (SNPs) in the promoter region of the cytotoxic T-lymphocyte antigen-4 (CTLA-4) gene are directly associated with the progression and onset of various human cancers. These SNPs are important prognostic biomarkers for the prediction and early onset of cancer risk. The variant frequency of the CTLA-4 rs11571317 (−658 C/T) polymorphism may be affecting the various ethnic groups differently. In the present study, the allelic frequency distribution of −658 C/T polymorphism was assessed in the population of Saudi Arabia and compared with other world populations. The data from different cancers were extracted from case-control studies in the various ethnic groups by using PubMed (MEDLINE) and similar web databases. The frequency of CTLA-4 rs11571317 (−658 C/T) variant allele (T) was observed to be 25.5% and different frequencies were found significant for India (p = 0.001), USA (p = 0.03), and China (p = 0.04), when the prevalence of Saudi Arabian population was compared to that of other population groups. The current finding reveals that there is a distinct pattern of CTLA-4 rs11571317 (−658 C/T) polymorphism variant allele in the populations of Saudi Arabia, may be because of the differences in ethnicity. The observed findings can help in the assessment of the risk for the population harboring the risk allele of rs11571317 (−658 C/T) SNP and toward their subsequent susceptibility to cancer.

## Introduction

Cancer is a multifactorial genetic disease and is one of the important causes of death in the world, with about 10 million reported deaths in 2020 [1,2]. Cancer is a problem of global health in the current era and also an important factor of death throughout the world. It is progressed by the overgrowth of abnormal cells, which are mutated from tissues of the normal cells. These groups of cells grow in a disorderly manner, have high metabolism, rapidly divide, and establish certain properties like colonizing new sites. Such types of properties and behavior of cancer cells have an evolutionary outcome in comparison to normal cells. Such abnormal overgrowth can kill cells by preventing the normal functioning of vital organs or spreading all over the body (metastasis) through the blood and lymph, damaging essential systems.

Colorectal cancer (CRC) results from the combination of epigenetic and genetic modifications. The transformation of epithelial cells into colorectal adenocarcinoma can be caused by several biological pathways. CRC represents one of the major causes of cancer-related mortalities in Western countries and, along with lung, prostate, and breast cancer, is among the most common malignancies [3,4]. The causes of cancers are some external factors (chemicals, tobacco, radiation, and infectious agents) and various internal factors (hormones, immune conditions, genetic mutations, and metabolic mutations). These causative factors act simultaneously leading to the initiation or promotion of carcinogenesis. The chemical substances found in the environment with carcinogenic properties have a direct or indirect influence on the cytoplasmic and nuclear region of cells leading to genomic disorders and genetic mutation [5]. Other carcinogenic factors are bacteria, viruses, and various radiations that comprise approximately 7% of all types of cancers. Globally, cancer is an important risk factor for mortality [6]. It is estimated that approximately 14.1 million people in 2012 were diagnosed with cancer, and it was reported that around 8.2 million deaths occurred worldwide. The incidence of new cases of cancer and their fatalities due to the rise of cancers was very sharp in the year 2018, i.e., 18.1 and 9.6 million, respectively [7]. In the case of men, the higher percentage of different types of cancers is in the order of the prostate, bronchus and lungs, rectum and colon, and urinary bladder. In the case of women, the occurrence of cancer is found to be higher in the order of the breast, bronchus and lung, colon and rectum, uterine, and thyroid. From these data, it is evident that prostate cancer in men and breast cancer in women contribute to a major part of the cancer [8].

The survival rate of cancer patients having unresectable tumors can be extended by the application of chemotherapy as well as targeted radiation therapy, although their adverse effects and resistance to drugs are considered challenging because of the heterogeneous nature of tumors [9]. Several studies have reported that there is a comprehensive correlation between cancer and the immune system, which lets down the basis of treatment by the application of immunotherapy [10].

The specialized mechanism of surveillance of the immune system enables controlling the growth of different types of tumors. The regulation of immune responses by co-stimulation and co-inhibition of signals is very important for cancer progression control. CTLA-4 is the most studied important checkpoint molecule in cancer progression [11]. The immunoglobulin CTLA-4 or CD152 expresses on the cell surfaces of T lymphocytes and works as activation of T-cells by negative regulation. The binding affinity of CTLA-4 has costimulatory receptors B7-1 (CD80) and B7-2 (CD86) over the antigen-presenting cells (APC), which produces an inhibition of signals that suppresses the activations of T cells [12]. It is evident from research that the CTLA-4 expression by cells of different types of tumors reduces antitumor immunity via T-cell mediation. Blocking of CTLA-4 controls the regulation of the anti-tumor immunity responses and induces autoimmune diseases.

The chromosomal localization of the gene CTLA-4 is at chromosome 2q33; this region has various genes associated with immune regulation [13]. This gene is highly polymorphic and various single nucleotide polymorphisms (SNPs) of this gene have effects on protein activities and expression of the gene. The activities like expression of genes and conformation of mRNA may be affected by these polymorphisms and their location, and thus it may have a direct association with the disease of human beings [14]. However, various studies reported that the polymorphisms of the CTLA-4 gene have a direct association with different diseases in diverse populations.

Earlier studies have reported that the polymorphisms of the CTLA-4 gene are associated with diseases of autoimmunity like hepatic cancer, Graves’ disease, kidney disease, breast cancer, vitiligo, systemic lupus erythematosus, and type I diabetes [15,16].

It was found that the polymorphism −658 C/T has an association with breast cancer in the Chinese Han population [15], although the importance of this polymorphism's functionality is not very well known. The CTLA-4 gene polymorphism −658C/T (rs11571317) is found in the promoter region, which induces the pattern of alternative splicing aberrantly and may alter the pattern of gene expression [17], leading to the predisposition of cancer progression.

Several epidemiological studies were conducted for the investigation of the correlation of the CTLA-4 rs11571317 −658 C>T polymorphism and their chances of cancer progression [18]. Although CTLA-4 is localized in the promoter region for the risk of cancers, their presence, and functional effects of CTLA-4 rs11571317 −658 C>T polymorphisms in the population of Saudi Arabia in any types of cancers have not yet been elucidated. The present study aims to determine the frequency distribution of the CTLA-4 rs11571317 −658 C>T polymorphisms among the healthy normal population of Saudi Arabia compared with various epidemiological investigations conducted around the world.

## Materials and methods

Criteria of search of gene variants

The databases were being searched from PubMed, EGEMS, and Web of Science for articles having keywords “CTLA-4,” “rs11571317,” “−658 C > T,” and “polymorphism.” The criteria of the search covered all the subjects of humans and was written in any language. Studies having the frequencies of genotypes of the population of the control group were included; however, studies containing frequencies of allele only and not having frequencies of genotypes were not included.

The name of the first author, publication year, nationality of the subjects, type of research, number of controls, criteria of inclusion and exclusion, and alleles and genotype frequencies of the subjects were abstracted for each study qualifying those criteria. If there were various reports found from the same race, the most recent published data were included. The data on the population of Saudi Arabia were included in the most recent reports.

In this study, the prevalence rate of CTLA-4 rs11571317 −658 C>T polymorphisms was extracted from six studies [15,18-22] (as shown in Table 1) and it was compared with the population of Saudi Arabia (as shown in Table 2) [18].

**Table 1:** Studies included in the CTLA-4 rs11571317 (−658 C/T) gene variant analysis in different populations CRC, colorectal cancer; BC, breast cancer; MM, metastatic melanoma; NMSC, non-melanoma skin cancer.

S. No.	Study	Country/ ethnicity	Race	Disease/ cancer types	Total no. of subjects (n)
1.	Al-Harbi et al. (2023) [18]	Saudi Arabia	Asian	CRC	90
2.	Ighid et al. (2022) [19]	Morocco	African	BC	200
3.	Queirolo et al. (2017) [20]	Italy	Europe	MM	100
4.	Goske et al. (2017) [21]	India	Asian	BC	285
5.	Welsh et al. (2009) [22]	USA	Caucasian	NMSC	786
6.	Wang et al. (2007) [15]	China	Asian	BC	148

**Table 2:** Observed and expected genotypic frequencies of CTLA-4 rs11571317 (−658 C/T) polymorphism in the control group of the study HWE, Hardy-Weinberg equilibrium; P-value, probability value; MAF: minor allele frequency.

Study	Observed genotype (n)			Expected genotype (n)			MAF	p-value (HWE) 18.12
	CC	CT	TT	CC	CT	TT		
Al-Harbi et al. (2023) [18]	61	21	14	53	36	06	0.255	0.00
* P-value < 0.05 was considered significant								

Statistical analysis

For the comparison of the genotypic and allelic frequencies, Pearson's χ2 test was applied to different populations by applying the SPSS statistical software program version 21 (IBM Corp., Armonk, NY). The Hardy-Weinberg equilibrium (HWE) was applied in this study by using Court Lab (a software program based on the web). P-value ≤ 0.05 was considered significant statistically.

## Results

According to the genotype distribution, the minor allele frequency (MAF) of CTLA-4 rs11571317 −658 C>T polymorphism in the population of Saudi Arabia was found to be 25.55%, which was consistent with the HWE accordingly, as shown in Table 2. The different MAFs were found in the genotype (CC, CT, and TT) and the distribution of allelic frequencies of the studied polymorphisms in various populations, as shown in Table 3. The MAF refers to the lower allele frequency, and it can vary by population. In association-based studies, MAF is widely used because it provides information to differentiate between common and rare variants in the population. The different MAFs were observed significantly for the ethnicity of India (p = 0.001), the United States (p = 0.03), and China (p = 0.04) to that of CTLA-4 rs11571317 −658 C>T observed frequencies in the Saudi Arabian populations and were compared to that of other population groups (p = 0.000).

**Table 3:** CTLA-4 rs11571317 (−658 C/T) gene variant genotype and allele frequency distribution in different populations and p-values in contrast to the Saudi Arabian population CRC, colorectal cancer; BC, breast cancer; MM, metastatic melanoma; NMSC, non-melanoma skin cancer; P-value, probability value; MAF, minor allele frequency.

S. No.	Study	Disease/tumor type	Total no. of subjects (n)	Genotype			Allele		Total alleles	C allele frequency	T allele frequency	p-value	MAF
				CC	CT	TT	C	T					
1	Al-Harbi et al. (2023) [18]	CRC	96	61	21	14	143	49	192	0.745	0.255	Ref.	25.55
2	Ighid et al. (2022) [19]	BC	200	148	44	08	340	60	400	0.85	0.15	0.09	15.0
3	Queirolo et al. (2017) [20]	MM	100	76	23	01	175	25	200	0.875	0.125	0.45	12.5
4	Goske et al. (2017) [21]	BC	285	39	246	00	324	246	570	0.568	0.432	0.001*	43.2
5	Welsh et al. (2009) [22]	NMSC	786	679	99	08	1457	115	1572	0.927	0.073	0.03*	7.3
6	Wang et al. (2007) [15]	BC	148	107	41	00	255	41	296	0.861	0.139	0.04*	13.9
* P-value < 0.05 was considered significant													

## Discussion

Cancer is a multifactorial disease, occurring due to the combination of genetics as well as various factors of the environment, which influence the progression of diseases during the lifetime. The cancer etiology is very complicated and is widely not known, although various factors like genetic as well as immunological play an important function. Networking of the immune system is a very complicated pathway of the biological system and their activation by antigens of tumors, which causes tumor cell destruction reported in some of the cases [23]. Natural killer cells and T-cells are involved in the rejection of tumors. The important elements of the adaptative immune system are CD8+ cytotoxic T-lymphocytes, which are employed in the defense of the host against the tumor cells.

The CTLA-4 is an important molecule of immuno-regulation that is involved in homeostatic mechanisms of the immune system, which is associated with the activation of T-cells and their tolerance. Apoptosis and cellular proliferation are the fundamental processes playing a vital function in developing tissue homeostasis [24]. Also, alteration in the apoptotic response may cause the accumulation of intimal cells by the process of therogenesis [25]. The genomic polymorphism of this molecule affects the level of transcription and translation leading to affecting their functions [21]. The existence of the association between the CTLA-4 gene variants and their splicing alternatively had been observed by Wang et al. (2007) [15].

It was observed in a recent study that the high CTLA-4 expression found in the cells of breast cancer is a prediction of the worst prognosis through antitumor immunity suppression. It is also reported from other studies that overexpression of CTLA-4 is about over 50% of breast tumor tissues in comparison to that of the tissues of benign breasts [26]. However, various studies demonstrated that the CTLA-4 molecule blockade urges antitumoral immunity and regresses tumors [27]. This finding supports the hypothesis that the involvement of CTLA-4 can be in the progression and developmental processes of cancer.

The polymorphic alleles of the CTLA-4 gene may be affecting the functions and levels of protein expressions [28]. However, various studies reported the association between the risk of breast tumors and CTLA-4 (−658 C/T) polymorphisms. The expression of CTLA-4 has distinct effects in the process of different phases of T-cell response, reaching the threshold for activation of T-cells, by suppressing the proliferation of T-cells and inducing apoptotic processes [29].

They may be directly contributing to regulating the B cell responses, although the expression of B-cells leads to the CTLA-4 after cell-cell connection with the activation of T-cells. It was observed that the polymorphisms of the CTLA-4 gene are associated with breast cancer in the populations of Iran [30].

The transcription factors binding site is found in the promoter region, which regulates the expression CTLA-4 gene [31]. It was reported for the first time that the CTLA-4 genotype located in the promoter −658 CC may increase breast cancer risks; however, the allele −658T has shown protective effects in breast cancers.

The SNP rs11571317 (−658 C>T) is linked to a higher risk of association with different cancers. However, genetic association studies have been used to discover common genetic variants or risk alleles (minor allele frequency > 10%) with small to moderate risks of cancer. MAF is widely used in population genetics studies because it provides information to differentiate between common and rare variants in the population.

Earlier studies have connected the rs11571317 gene to the cancer of colorectal, breast, metastatic melanoma, and non-melanoma skin cancer [15,19-22]. This is an association-based study; however, the association studies compare the frequency of a genetic variant in disease-affected patients and healthy controls. The SNP rs11571317 was observed as significantly associated with the populations of Saudi Arabia [18], confirming an early finding by Goske et al. (2017) [21], Welsh et al. (2009) [22], and Wang et al. (2007) [15].

In breast cancer cases, the prevalence rate of the rs11571317 (−658 C>T) polymorphisms showed a low susceptibility in Indians [21] and Chinese [15]. According to a study performed by Welsh et al. (2009) [22], the polymorphism rs7372209 C>T determines its impact on the US population's susceptibility to developing non-melanoma skin cancer.

The study conducted by Goske et al. (2017) [21] investigated the effect of polymorphism (rs11571317) found in the promoter region that is susceptible to breast cancer progression. It was found that the genotype CC of rs11571317 imparted a three-fold increase in the risk of breast cancer; however, in the genotype CT, a heterozygote was found as shielding. These findings were supported by a study conducted by Wang et al. (2007) [15], who reported that the genotype CC of this polymorphism found in the promoter region increases the risk of breast cancer, and the allele “T” was to be protective in the Han Chinese population.

The discrepancy among various studies is attributed to factors such as the assessed populations being of different ethnic groups, various methods of genotypes may affect the results, several studies have deviated from the HWE, and study design and methods for each study being different, leading to a reduction in consistency. However, cancers and other diseases of humans have different patterns of inheritance.

The progression of diseases and their consequences on genetic and epigenetic alterations, copy number variation, influences of various environmental factors, and the different prevalence of this rs11571317 (−658 C>T) SNP among the populations suggest the factors of susceptibility having various effects on the different population groups.

In this analysis, the frequencies of allele and genotype studied do not show the overall ranges of variants at the same location. Considering the genetic variants of CTLA-4 rs11571317 (−658 C>T) and its role in the maintenance of immunological balance, it has the potential to modify the immune responses and susceptibility to the development of cancer.

Moreover, this type of study provides insight into the developmental process of clinical and epidemiological databases for future prospects. It was identified by the genome-wide association studies (GWAS) and association of genetic studies that the common type of alleles and their frequencies contribute toward the component of hereditary for most of the prevalent complex diseases.

The association of several genetic tests and different genetic models is needed for the identification of essential genes and their corresponding SNPs participating in the progression and development of early therapeutic preventions of the disease leading to the possible treatments of cancer [32].

The biological importance and understanding of such types of genetic variables would be needed for the discovery of SNPs that affect the clinical outcomes or susceptibility traits in various population groups.

However, there are several shortcomings, like the application of statistics and computational analysis, and also the reproducibility factors that must be considered for essential genetic markers applied in the associations of the gene-disease research that could be identified [33]. Our study is based on a limited number of subjects, and more data are required, which will be helpful for further research in the future.

## Conclusions

The allelic variants of rs11571317 (−658 C>T) polymorphism in the population of Saudi Arabia substantially vary from different populations on the globe. The observed findings can be applied to the screening of colorectal cancer in the populations, as well as in the assessment and predisposition of disease progression significantly, and can be used as a potential biomarker for cancer progression.
